# A high-resolution phase-contrast microscopy system for label-free imaging in living cells

**DOI:** 10.1247/csf.24018

**Published:** 2024-05-25

**Authors:** Kentaro Shimasaki, Yuko Okemoto-Nakamura, Kyoko Saito, Masayoshi Fukasawa, Kaoru Katoh, Kentaro Hanada

**Affiliations:** 1 Department of Biochemistry and Cell Biology, National Institute of Infectious Diseases, Shinjuku-ku, Tokyo 162-8640, Japan; 2 Biomedical Research Institute, National Institute of Advanced Industrial Science and Technology (AIST), Tsukuba-shi, Ibaragi 305-8566, Japan; 3 AIRC, National Institute of Advanced Industrial Science and Technology (AIST), Koto-ku, Tokyo 135-0064, Japan; 4 Center for Quality Management Systems, National Institute of Infectious Diseases, Shinjuku-ku, Tokyo 162-8640, Japan

**Keywords:** label-free imaging, organelle dynamics, virus infections, apodized phase contrast

## Abstract

Cell biologists have long sought the ability to observe intracellular structures in living cells without labels. This study presents procedures to adjust a commercially available apodized phase-contrast (APC) microscopy system for better visualizing the dynamic behaviors of various subcellular organelles in living cells. By harnessing the versatility of this technique to capture sequential images, we could observe morphological changes in cellular geometry after virus infection in real time without probes or invasive staining. The tune-up APC microscopy system is a highly efficient platform for simultaneously observing the dynamic behaviors of diverse subcellular structures with exceptional resolution.

## Introduction

The phase-contrast microscope, a widely-used optical device among cell biologists, provides high-contrast images in unstained cells compared to the brightfield microscope (Zernike, 1942a, 1942b). However, it presents an issue with halo artifacts, which are spurious bright areas surrounding phase objects, particularly problematic when observing subcellular structures in detail. The light diffracted by larger phase objects creates more substantial halos that obscure smaller subcellular structures around them and degrade image quality in phase-contrast microscopy (Otaki, 2000; Pelc *et al.*, 2020). The apodization technique was introduced to address this challenge: apodization annuli, acting as neural density filters, are strategically positioned around the phase ring within the objective lens. These annuli selectively reduce the transmittance of diffractive light emitted by larger phase objects. As a result, this reduction effectively minimizes the presence of halo artifacts, enhancing the overall quality of phase images (Otaki, 2000; Pelc *et al.*, 2020).

Here, to establish a label-free imaging platform that can capture the dynamic processes of subcellular structures in unstained living cells, we provide procedures to adjust a commercially available apodized phase-contrast (APC) microscopy system with several modifications. The tuned-up APC microscopy system allowed us to obtain high-resolution phase-contrast digital images without any image reconstruction.

## Materials and Methods

### Cell culture

The African green monkey kidney cell line Vero cells (JCRB9013) were maintained in Eagle’s minimum essential medium (EMEM) supplemented with 5% heat-inactivated fetal calf serum (FCS) and penicillin-streptomycin. The HeLa mCAT#8 cell line, established as described elsewhere (Yamaji *et al.*, 2010), was used as the HeLa wild-type cell line. HeLa cells were cultured in high-glucose D-MEM supplemented with 10% heat-inactivated FCS and penicillin-streptomycin. Cells were grown at 37°C in a 5% CO_2_ incubator. Before live-cell imaging, cells were seeded on glass bottom dishes (#3970-035, #3971-035, or #3922-035, AGC TECHNO GLASS CO., LTD., Haibara-gun, Shizuoka, Japan) in the medium specific for live-cell imaging: For Vero cells, phenol red-free EMEM (#05901, Nissui Pharmaceutical Co., LTD., Tokyo, Japan) supplemented with 5% heat-inactivated FCS, 2 mM L-glutamine, and 2.2 g/L sodium hydrogen carbonate, and for HeLa cells, FluoroBrite D-MEM (A1896701, Thermo Fisher Scientific, Waltham, MA, USA) supplemented with 10% heat-inactivated FCS, 2 mM L-glutamine, and penicillin-streptomycin.

### Establishment of stable cell lines expressing fluorescent organelle markers

Vero cells were transfected with the plasmids that encode the respective fluorescent organelle markers using the FuGENE6 transfection reagent (E2691, Promega, Madison, WI, USA), following the manufacturer’s protocol. After 2 days of transfection, cells were re-seeded and cultured in the presence of 1 mg/mL G418 for 7 days. After G418-selection, cells transfected with pAcGFP1-Mito (#632432, Clontech, Mountain View, CA, USA), pAcGFP1-Endo (#632490, Clontech), pAcGFP1-Golgi (#632418, Clontech), pAcGFP1-F (#632511, Clontech) or pER-mAG1 (AM-V0202M, MBL, Tokyo, Japan), and exhibiting fluorescent signals were isolated by FACS cell sorter and further subjected to single-cell cloning.

### Viruses and infection

Vero cells were seeded at a density of 1 × 10^5^ cells/ dish in a glass base dish (#3971-035, AGC TECHNO GLASS CO., LTD.) one day before inoculation. The cells were infected with the Japanese encephalitis virus (JEV) rAT strain (Zhao *et al.*, 2005) at a MOI of 1.0 or 2.1 at 37°C for 2 h in 1% FCS-EMEM, and the medium was replaced with the fresh normal culture medium.

### Immunocytochemistry

Cells were seeded onto a glass bottom dish and fixed with 4% paraformaldehyde (PFA) at room temperature for 15 min, followed by ice-cold methanol for 3 min. The cells were then blocked with 5% heat-inactivated FCS in phosphate-buffered saline (PBS) for 30 min. To detect non-structural protein NS3 of JEV and double-stranded RNA (dsRNA), cells were incubated with a rabbit polyclonal anti-NS3 antibody (GTX125868, GENETEX, Irvine, CA, USA, diluted at 1:500) and a mouse monoclonal anti-dsRNA antibody (#10050300, English & Scientific Consulting Kft., Szirák, Hungary, diluted at 1:500) for 1 h, followed by incubation with anti-rabbit donkey IgG H&L DyLightR 488 (ab96919, Abcam, Cambridge, UK, diluted at 1:1000) and anti-mouse goat IgG H&L DyLightR 550 (ab96880, Abcam, diluted at 1:1000) for 1 h at room temperature. To detect the endoplasmic reticulum (ER) luminal proteins bearing tetrapeptide KDEL, cells were fixed with 4% PFA at room temperature for 15 min and permeabilized with 0.1% Triton-X in PBS for 5 min, and then blocked with 5% heat-inactivated FCS in PBS for 30 min. Cells were incubated with a mouse monoclonal antibody against KDEL (M181-3, MBL, diluted at 1:800), followed by anti-mouse donkey IgG H&L DyLightR 488 (ab96871, Abcam, diluted at 1:1000). After washing with PBS, the glass bottom dish with fixed cells was filled with PBS and the cells were subjected to imaging.

### Microscopic observation

A day before imaging, cells (1 × 10^5^ cells/dish) were seeded on a glass base dish. To label lysosomes, lipid droplets, or the ER, cells were incubated for 30 min with 75 nM LysoTracker Green (LTG; L7526, Thermo Fisher Scientific), for 30 min with 1 μM LipiDye II (LD II; FDV-0027, Funakoshi, Bunkyo-ku, Tokyo, Japan), or for 15 min with 1 μM ERseeing (FDV-0038, Funakoshi), respectively. After labeling, the medium was replaced with fresh phenol red-free medium.

The APC microscopy system consisted of an inverted microscope (ECLIPSE Ti2; MEA54000, Nikon, Tokyo, Japan), a 100× objective lens with an apodized phase plate (Plan Fluor ADH 100×/NA 1.30 Oil; MRH41902, Nikon), and NIS-Elements AR software (MQS31000, Nikon) to control image acquisition. For transmitted illumination, a narrow band pass filter (546 ± 3 nm; FF01-546/6-45-D, Semrock, Rochester, NY, USA) was placed in the optical light path to make monochromatic green light from a white high-power LED (TI2-D-LHLED, Nikon). For epi-fluorescence illumination, a multi-wavelength LED illumination system (X-Cite TURBO, Excelitas Technology Corp., Waltham, MA, USA) was employed. To detect fluorescent signals of AcGFP1, monomeric Azami-Green1 (mAG1), LTG, LD II, ERseeing, and DyLightR 488, 475 nm LED light was used with a fluorescent filter cube, GFP Basic C-FL (MXK38474, Nikon). To image the fluorescence signal of DyLightR 550, 575 nm LED light was used with a filter cube, TRITC Basic C-FL (MXK38477, Nikon). It is important to note that when comparing the appearances of the same objects between APC and fluorescence images using non-apochromatic objectives such as the ADH objective, it is highly recommended to utilize green fluorescence. This is due to the potential inconsistency in fluorescence wavelength compared to monochromatic transmitted green light, which can result in chromatic aberrations in the two images. These aberrations particularly affect the appearances of smaller and thinner structures, such as the tubules of the peripheral ER. To achieve near-simultaneous acquisition (time lag: 400–500 milliseconds) of APC and fluorescence images in living cells, a GFP Basic C-FL filter cube was kept in place even during transmitted illumination. This reduced filter-changing times and minimized the time lag between imaging with the two modalities. All images were recorded by a back illumination type sCMOS camera (ORCA-Fusion BT, Hamamatsu Photonics K.K., Hamamatsu-shi, Shizuoka, Japan) under the same exposure time and LED power for each type of image, and exported as 16-bit ND2 files (2304 × 2304 pixels). All the imaging experiments in living cells were performed under the conditions of 37°C and 5% CO_2_ using the stage top incubator (STXG-WSKMX-SET, Tokai Hit., Co, Ltd., Fujinomiya-shi, Shizuoka, Japan) with the adaptor (TI2-ZILCS, Tokai Hit., Co, Ltd.). During time-lapse imaging, the Z-position was controlled using the Perfect Focus System (Nikon) to prevent focus drift. It’s worth noting that although we utilized this APC microscopy system in a previous study (Saito *et al.*, 2022), detailed information about the system was not provided in that study.

### Image processing and analysis

The exported ND2 files were converted to 16-bit grayscale TIFF files using Fiji (Schindelin *et al.*, 2012) and subject to the following image processing. To mitigate the effect of non-uniform intensity (optical noise caused by optical components in the light path) on the background, all the APC images underwent background subtraction. Raw APC images were subtracted by the background image, which was generated by averaging the background image stack (Z-stack slices with 0.1 or 0.3 μm steps ranging to 6 μm). Subtraction of the APC images was performed in 32-bit float, and the outputs were converted to 16-bit images. Fluorescent images were deconvoluted using NIS-A 2D/3D deconvolution modules (Richardson-Lucy method; MQS42700, Nikon) loaded into the NIS-Elements software. The contrast of each image of the same condition was adjusted to the same setting based on a look-up table using the ‘Enhance Contrast’ of Fiji.

In Fig. 3D–E, to quantify the ratios of the areas of the dark puncta inside the nucleus to the areas of the nucleus themselves, a pixel classifier was trained with the dark structures inside the nucleus including nucleoli in the APC images of the JEV-infected Vero cells, and non-infected cells, using Ilastik software version 1.40 (Berg *et al.*, 2019). The outputs were exported as described above. The ROIs in areas of the nucleus were manually acquired. The automated analysis with CellProfiler software version 4.2.6 (Stirling *et al.*, 2021) was conducted, using the CP module-pipelines as follows:

*MaskImage* was used to remove the binary masks of the segmented results outside the nuclei to be analyzed.

*ConvertImageToObjects* for the binary ROI image of the nuclei.

*ConvertImageToObjects* for the binary ROI image of the segmented results to be masked above.

*RelateObjects* was used to associate the segmented structures to parent nuclei.

*SplitOrMergeObjects* was used to merge the individual objects of the segmented structures as one object.

*MeasureObjectSizeShape* was used to measure the areas of the nuclei and the segmented structures.

*CalculateMath* was used to add the calculation result of the ratios of the areas of the segmented structures to these of the nuclei.

*ExportToSpreadSheet* for exporting the results as CSV files.

The analyzed results were visualized using Matplotlib and Seaborn libraries in Python.

### Statistical analysis

Statistical analyses were conducted by EZR software (Kanda, 2013), which is a graphical user interface for R (The R Foundation for Statistical Computing, Vienna, Austria). The Student’s *t*-test was carried out for two-group comparisons. All *p*-values were two-sided and *p*-values of 0.05 or less were considered statistically significant.

## Results and Discussions

### Amendments to the commercially available APC microscope

**Fig. 1:**
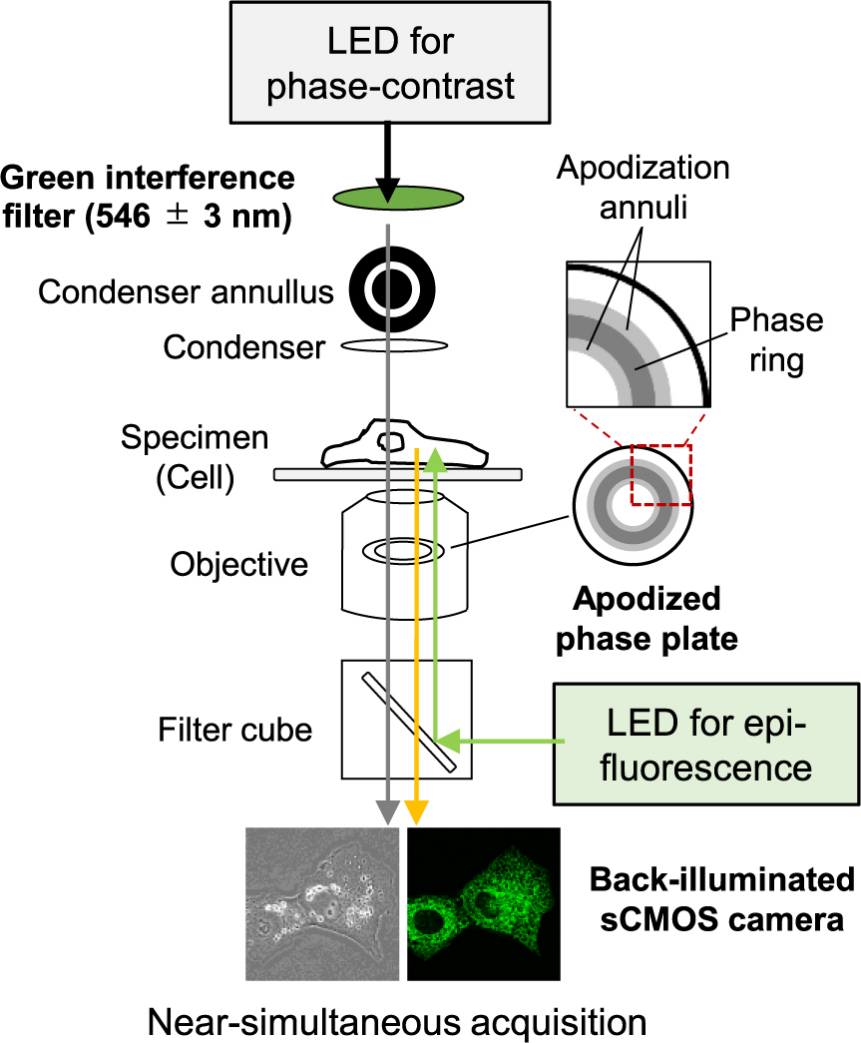
Schematic diagram of the tune-up APC microscopy system. This system is based on a conventional phase-contrast and epi-fluorescence microscopy setup, enhanced by the incorporation of an objective with an apodized phase plate. Apodization annuli, acting as neutral density filters, are placed around the phase ring to attenuate diffracted lights from larger phase objects, thereby reducing halo artifacts. In addition to utilizing this APC objective lens, this system incorporates trans-illumination via a narrow bandpass green interference filter and a back-illuminated sCMOS camera, contributing to the capturing of high-resolution APC images.

APC is a technique that reduces the halo effect observed around phase objects, allowing for the visualization of small structures hidden behind the halo. To enhance this capability, we have modified several microscope components (Fig. 1A) better to observe intracellular small compartments in unstained living cells.

#### 1. Trans-illumination using monochromatic light (546 nm):

The international standard (ISO 7944: 1988) has specified the mercury e-line 546,07 nm as the reference wavelength for non-ophthalmic applications. Since bright-field microscopy with transmitted light is most widely used, the same light source is shared for phase-contrast observation. Halogen lamps with a wide bandpass filter (546 ± 50 nm) or, more recently, white light-emitting diodes (LEDs) are commonly used for phase-contrast observations. These light sources may cause the degradation of phase-contrast images. To minimize this problem, we incorporated a narrow bandpass filter (546 ± 3 nm) in front of the light source (white LEDs).

#### 2. LED and fluorescent cube:

To achieve quick switching of illumination light, we have implemented LEDs for both trans-illumination (while LED + bandpass filter (546 nm)) and epi-illumination (488 nm), allowing us to switch between these light paths seamlessly: APC and fluorescence (488 nm), with the LEDs functioning in an ON/OFF manner. This enhancement has enabled optical path switching within 400–500 milliseconds without vibration.

#### 3. Subtraction of background images:

Phase-contrast images encompass phase differences derived from two factors: the samples (or cells) and the optical components in the light path. The latter introduces optical noise, which obscures genuine signals from the sample and degrades image resolution. To mitigate this effect, we employed post-processing on recorded images by subtracting the background images, which consist of images from the field of view with no cells. Removing optical noises through background subtraction can lead to detecting subtle phase retardation, even less than 1 nm (Katoh *et al.*, 1999).

#### 4. Back-illuminated sCMOS camera:

To capture images of small intracellular compartments, it is imperative to detect phase differences of less than 1 nm (Katoh *et al.*, 1999). Achieving this precision with a commercially available apodized phase-contrast objective lens (Plan Fluor ADH 100×/NA 1.30 oil) necessitates improving the signal-to-noise ratio of the images. The ADH objective lens reduces background light by 90%, resulting in high contrast but relatively low brightness. After exploring camera options to overcome this dilemma, we found that a back-illuminated sCMOS (scientific Complementary Metal-Oxide-Semiconductor) camera strikes the optimal balance between sensitivity and a high image recording rate. Additionally, this camera’s wide dynamic range allows for detecting subtle signals to high-power signals, maximizing the effectiveness of background subtraction and enabling the detection of phase differences among diverse intracellular structures.

### Visual characterization of various organelles in the APC images

**Fig. 2:**
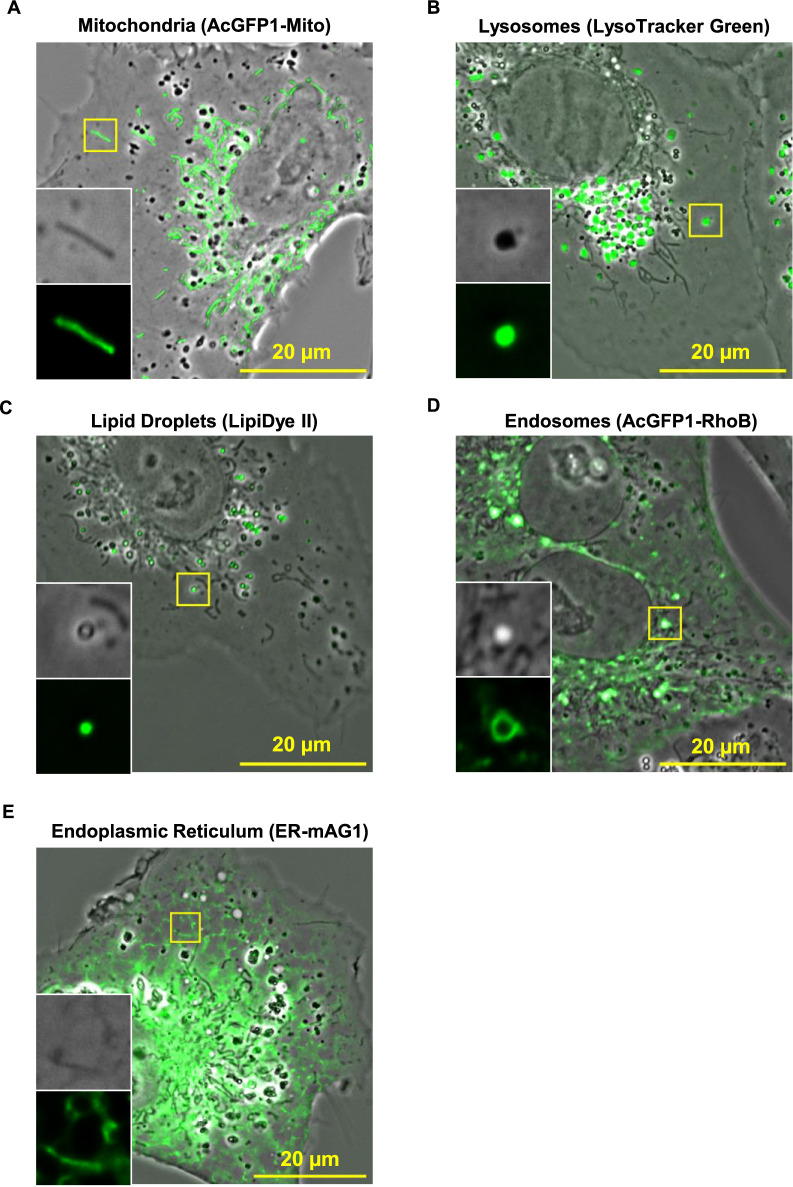
Visual characterization of various organelles in APC images. Vero cells were labeled with AcGFP1-Mito for mitochondria (A), LysoTracker Green for lysosomes (B), LipiDye II for lipid droplets (C), AcGFP1-RhoB for endosomes (D), and ER-mAG1 for the ER (E). The insets represent the enlarged areas of the yellow boxes with APC and fluorescence images. The scale bars in (A)–(E) are 20 μm. For detailed visual characterization using z-stack slices, see also Supplemental Materials Fig. 1A–E.

Due to the susceptibility to various viruses, African green monkey kidney-derived Vero cells have been widely used for research on infectious diseases (Osada *et al.*, 2014; Sakuma *et al.*, 2018). They display relatively flat morphology, which is advantageous for live imaging of intracellular structures. Thus, we employed Vero cells as a model cell type in this study. To determine which organelles the observed subcellular structures correspond to, we employed a near-simultaneous acquisition of APC and fluorescence images using organelles markers. Mitochondria were visualized as lint-like structures darker than cytosol (Fig. 2A, Supplemental Fig. 1A, and Video 1). The vesicular organelles represented each characteristic appearance; lysosomes were darker vesicles (Fig. 2B, Supplemental Fig. 1B, and Video 2), endocytic vesicles appeared to be brighter than cytosol (Fig. 2C, Supplemental Fig. 1C, Video 3 and Supplemental Fig. 2A–C), and lipid droplets were bright vesicles of which the edges were darker than the cytosol (Fig. 2D, Supplemental Fig. 1D and Video 4). In the case of the ER, the landmarks of APC images correlated with distributed reticular fluorescence signals were difficult to discern in the perinuclear regions due to interference from other structures (Fig. 2E). However, in the sparse peripheral areas, we could observe the reticular structures corresponding to the fluorescent signals of the ER, which appeared slightly darker than the cytosol in the APC images (Fig. 2E, Supplemental Fig. 1E and Video 5). Notably, no discernible structural features corresponding to the fluorescent signals were seen in the case of the Golgi apparatus (Supplemental Fig. 3). These image characteristics of organelles in APC images were also observed in other mammalian cell lines, such as HeLa cells (Supplemental Fig. 4). It is noteworthy that the peripheral ER exhibited lower contrast compared to those of Vero cells. This discrepancy may be attributed to the increased thickness of HeLa cells compared to Vero cells, which could potentially hinder the detection of subtle phase differences in the peripheral ER.

Even when the apodized objective lens (ADH) was replaced with a non-apodized phase-contrast objective lens having the same numerical aperture (Plan Fluor DLL 100×/NA 1.30 oil) in our microscopy system, various intracellular structures, including lint-like mitochondria (yellow arrowheads in Supplemental Fig. 5A), peripheral ER (ROI 2 and 4 in Supplemental Fig. 5A), and vesicles (magenta arrowheads in Supplemental Fig. 5A), were discernibly observed in living cells, although the ADH objective gave slightly clearer images than the DLL objective, possibly due to local halo reduction by apodization. Interestingly, the appearance of certain vesicular structures differed between the two lenses: vesicular structures appeared bright with darker edges when imaged with the ADH objective while they appeared black when imaged with the DLL objective (Supplemental Fig. 5A). This distinct image characteristic is not attributable to a blurred effect (Supplemental Fig. 5B), suggesting that the ADH objective has the potential to reveal diverse vesicular structures with unique characteristics more distinctly than the DLL objective. We opted for the ADH objective for subsequent analyses based on these advantages.

Essentially, the visual characteristics of phase-contrast images, including brightness and contrast pattern, are refractive of differences in refractive index and thickness of the specimen (Zernike, 1942a, 1942b). It is reasonable to assume that the appearance of these vesicular organelles is primarily influenced by variations in their refractive indices, as differences in thickness or diameter may be insufficient to account for the distinct appearances observed. Within cells, various biological macromolecules, such as proteins, lipids, and nucleic acids, contribute to the refractive index of phase objects, and organelles are formed as complex combinations of these biological components with local distributions. For instance, lysosomes contain a large number of luminal proteins involved in the substrate-degradation (hydrolases, its cofactors, or degraded proteins), and seem to be dense structures, compared to endocytic vesicles (Ballabio and Bonifacino, 2020; Huotari and Helenius, 2011), while lipid droplets possess a hydrophobic core of neutral lipids, which is surrounded with a phospholipid monolayer with a specific set of proteins (Olzmann and Carvalho, 2019). The variations in these biomolecular compositions and distribution create distinct local refractive index profiles, thereby exhibiting the unique visual characteristics of each organelle in APC images.

### Unstained live-imaging with Japanese encephalitis virus-infected cells

Infections of mammalian cells by biological pathogens such as viruses often lead to significant alterations in the subcellular structures of the host cells (Robinson *et al.*, 2018; Thakur *et al.*, 2019). The Japanese encephalitis virus (JEV), a single-stranded positive-sense RNA virus belonging to the Flavivirus genus of the Flaviviridae family, is a mosquito-borne zoonotic virus. Most JEV infections in humans are mild or asymptomatic, but approximately 0.4% result in severe encephalitis, causing approximately 13,000 to 20,000 deaths annually (Campbell *et al.*, 2011). Within the host cell cytoplasm, single-stranded positive-sense RNA viruses like JEV induce remodeling of intracellular membranes, giving rise to structures referred to as viral replication organelles. These induced organelles serve as platforms for the replication of the viral RNA genome (Wolff *et al.*, 2020).

**Fig. 3:**
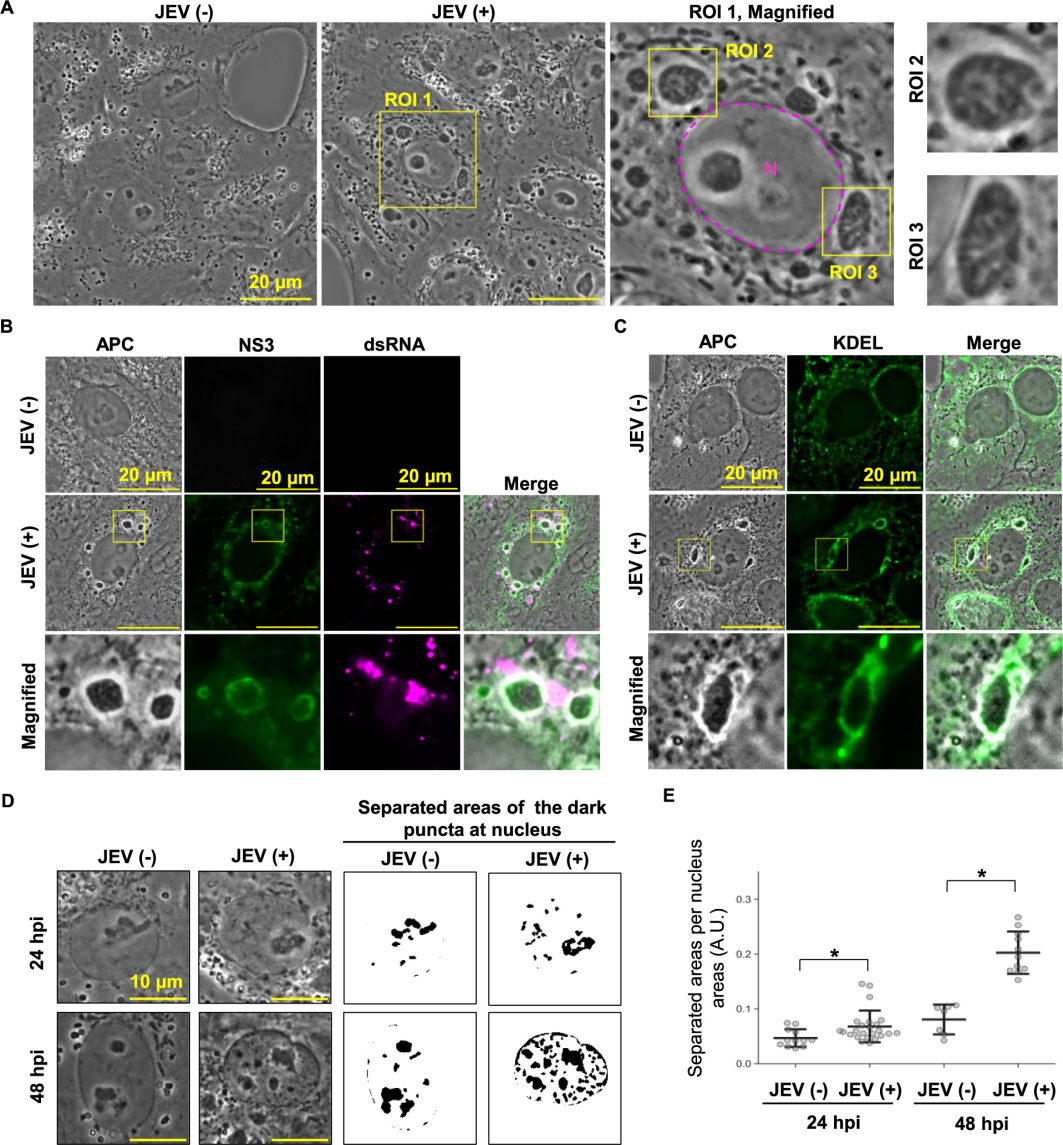
Unstained live-cell imaging in Japanese encephalitis virus (JEV)-infected cells. (A) Representative APC images of living cells infected with JEV at 24 h post-infection (hpi) (MOI 2.1) and non-infected cells. The ROIs (yellow boxes) represent the magnified areas. The ROI1 shows the JEV-induced structures like the convoluted membranes around the nucleus (magenta dotted lines). In further magnified ROI2 and 3, these structures have an uneven appearance in their brightness. (B) Representative images of JEV-infected cells stained with anti-NS3 viral protein antibody and anti-double stranded-RNA antibody. Cells were infected with JEV at MOI 1.0, and immunocytochemistry was performed at 24 hpi. The yellow boxes indicate the magnified areas of each image. (C) Representative images of cells stained with anti-KDEL antibody. Cells were infected with JEV at MOI 1.0, and immunocytochemistry was carried out at 48 hpi. The yellow boxes show the magnified area of each image. (D) Representative APC images of the nucleus in JEV-infected (MOI 2.1) or non-infected living cells. The right panels show the representative results of machine-learning-based segmentation of these punctate structures (including nucleoli) from each nucleus in the APC images. (E) Quantification of the dark and punctate structures at the nucleus in the APC images. Each dot shown indicates the ratio of the area of the separated structures to that of the whole nucleus in each cell. The medial bars are the means of the ratios and the upper and lower bars indicate the standard deviation (7–24 cells were analyzed). Two independent experiments were carried out and similar results were obtained. *: p<0.05, ns: not significant (Student’s t-test). The scale bars in (A)–(C) are 20 μm, while in (D) are 10 μm.

To determine whether the tuned-up APC microscopy system could be a powerful tool for analyzing infection-dependent changes in host cell structures without the use of probes, we observed cells infected with JEV. After JEV infection, significant morphological changes in intracellular structures were evident when compared to uninfected control cells. Notably, large dark oval structures, resembling the virus replication organelles known as convoluted membranes (CMs) (Ci and Shi, 2021; Morita and Suzuki, 2021), were observed in the perinuclear regions (Fig. 3A). To confirm the identity of these observed structures as CMs, immunocytochemistry was performed using fixed cells. The fluorescence signals of NS3, a non-structural viral protein with ATPase and RNA helicase activities (Yamashita *et al.*, 2008), corresponded to the periphery of the CMs-like structures seen in the APC images (Fig. 3B). Additionally, signals from the KDEL ER marker exhibited a similar pattern to NS3 (Fig. 3C). Furthermore, signals from double-stranded RNA (dsRNA), which formed as an intermediate in viral RNA replication, were enriched around these structures (Fig. 3B). These findings align with previous studies demonstrating that flavivirus-induced CMs originate from the ER with the viral proteins and that viral replication takes place around these structures (Yu *et al.*, 2017; Tabata *et al.*, 2021), thus strongly suggesting that the dark oval structures observed in the APC images are the JEV-induced CMs.

In addition to the formation of CM-like structures, we also found distinct features in JEV-infected cells. At 48 h post-infection (hpi) there was an apparent increase in dark puncta within the nucleus, compared to observation at 24 hpi (Fig. 3D). A quantitative analysis using a machine-learning-based pixel classifier confirmed that the JEV infection induced the formation of these structures at 48 hpi, in addition to the slight increase in these puncta at 24 hpi (Fig. 3E). Furthermore, at 24 hpi, the emergence of seemingly bulky structures/areas around the perinuclear regions was observed (yellow arrowheads in Supplemental Fig. 6). Time-lapse imaging revealed that these structures appeared relatively static compared to other motile structures such as vesicles and mitochondria-like structures (Video 6). These findings provided dynamic insights into subcellular structures in JEV-infected living cells under unstained conditions.

## Conclusions

The tuned-up APC microscopy system is a powerful tool that allows us to observe the dynamic behaviors of various organelles in living cells without the need for staining or invasive procedures. It provides a resolution high enough to distinguish several organelle types distinctly. This system proves to be convenient when simultaneous observation of multiple subcellular compartments is necessary, especially for analyzing physical interactions and distributions between different organelles (Scorrano *et al.*, 2019). Notably, the APC microscopy system is particularly useful in studying and categorizing morphological changes induced by pathogen infections in host cells, without requiring any genetic modifications or invasive treatments of biological specimens.

More precise quantitative analyses of the APC images require segmentation of biological objects such as organelles in this panoramic image. To facilitate this necessary but laborious task, artificial intelligence technology, including a supervised type of deep learning exploiting fluorescent marker images as the origin of training data, can accelerate and improve the accuracy and objectivity of the segmentation process in the near future. This potential for improved segmentation using deep learning can make the APC microscopy system an even more beneficial tool in the study of cellular structures and functions.

To date, several types of imaging approaches employing computational imaging techniques for high-resolution monitoring of subcellular structures in unstained living cells have been developed in optical microscopy, including the differential interference contrast and polarized microscope (Ordenbourg, 1996; Shribak and Inoue, 2006), as well as more recently, the phase-contrast microscope (Hugonnet *et al.*, 2022; Ma *et al.*, 2022; Otaki and Katoh, 2023). These methods generate a single high-resolution image through reconstruction from several raw images acquired under different illumination conditions in each unique optical setup. In the case of the APC microscopy system, fine-contrast phase images are obtained through optical techniques such as apodization and monochromatic light illumination. Importantly, the improved panoramic images are directly recorded on a digital camera without the need for any subsequent image reconstruction.

## Data Availability

We deposited the uncompressed videos, raw images in each processing step, and analyzed data in J-STAGE Data (doi: 10.51021/data.csf.25824388).

## Funding

This work was financially supported in part by MEXT KAKENHI (No. JP17H06417), JSPS KAKENHI (JP21H02630), and AMED (JP22fk0108561j0101) to K.H.; MEXT KAKENHI (No. JP17H06413) to K.K.; AMED (JP20he0622012) to K.K. and K.H.

## Data Availability

The supporting information for this article is available in J-STAGE Data.

## Abbreviations

APC: apodized phase contrast
CM: convoluted membrane
dsRNA: double stranded RNA
ER: endoplasmic reticulum
hpi: hours post-infection
JEV: Japanese encephalitis virus
LD II: LipiDye II
LED: light-emitting diode
LTG: LysoTracker Green
ROI: region of interest
sCMOS: scientific Complementary Metal-Oxide-Semiconductor)

## Supplementary Material

Video 1 Time-lapse movie of an area in a Vero cell expressing AcGFP1-Mito detected by APC microscopy, related to Fig. 2A. Whole-cell images were captured at 3-second intervals over a 1-minute 45-second duration. The timestamp at the upper left corner indicates minutes:seconds. The images, presented in the order of APC, AcGFP1-Mito, and merged views, progress from left to right. The scale bar indicates a length of 20 μm. This movie was created from the original TIFF stacks through compression into JPEG format, followed by conversion into MP4 files.

Video 2 Time-lapse movie of an area in a Vero cell stained with LTG detected by the APC microscopy, related to Fig. 2B. Whole-cell images were recorded at 3-second intervals over a 2-minute 12-second duration. The timestamp at the upper left indicates minutes:seconds. The images, presented in the order of APC, LTG, and merged views, progress from left to right. The scale bar represents 20 μm. This movie was created from the original TIFF stacks by compression into JPEG format, followed by conversion into MP4 files.

Video 3 Time-lapse movie of an area in a Vero cell stained with LD II detected by the APC microscopy, related to Fig. 2C. Whole-cell images were captured at 3-second intervals over a 2-minute 15-second duration. The timestamp at the upper left represents minutes:seconds. The images, presented in the order of APC, LD II, and merged views, progress from left to right. Scale bars are 20 μm. This movie was created from the original TIFF stacks through compression into JPEG format, followed by conversion into MP4 files.

Video 4 Time-lapse movie of an area in a Vero cell expressing AcGFP1-RhoB detected by APC microscopy, related to Fig. 2D. Whole-cell images were recorded at 3-second intervals over a 2-minute 18-second duration. The timestamp at the upper left indicates minutes:seconds. The images, presented in the order of APC, AcGFP1-RhoB, and merged views, progress from left to right. The scale bar represents 20 μm. This movie was created from the original TIFF stacks through compression into JPEG format, followed by conversion into MP4 files.

Video 5 Time-lapse movie of an area in a Vero cell expressing ER-mAG1 detected by the APC microscopy, related to Fig. 2E. Whole-cell images were captured at 3-second intervals over a 3-minute duration. The timestamp at the upper left indicates minutes:seconds. The images, presented in the order of APC, ER-mAG1, and merged views, progress from left to right. The scale bar represents 20 μm. This movie was created from the original TIFF stacks through compression into JPEG format, followed by conversion into MP4 files.

Video 6 Time-lapse movie of areas in Vero cells infected with JEV detected by the APC microscopy, related to Fig. 3A. Whole-cell images were recorded at 0.2-second intervals over a 9.8-second duration. An area of the uninfected Vero cell (left panel), and two areas of the JEV-infected cells (middle and right panels) are shown. The scale bar represents 10 μm. This movie was created from the original TIFF stacks through compression into JPEG format, followed by conversion into MP4 files.
