# Supplementary material for: A high-resolution phase-contrast microscopy system for label-free imaging in living cells
Source: Cell Struct Funct. 2024 May 25;49(1):21–9. doi: 10.1247/csf.24018 (PMC11496782; doi:10.1247/csf.24018)
Supplement: Supplementary file 7 — Supplementary Materials [file csf_49_24018_7.pdf]

Supplemental Figure 1.

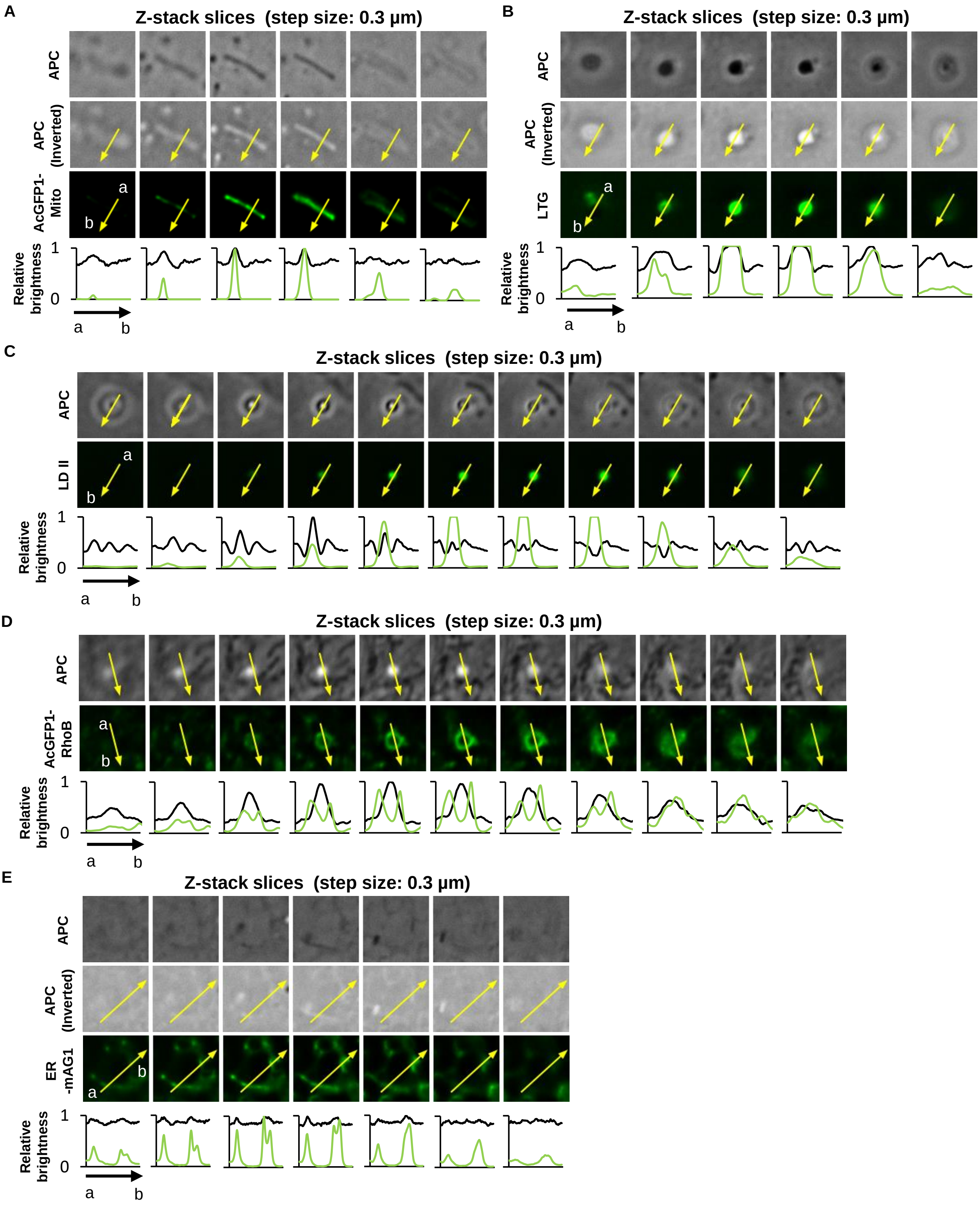

Supplemental Figure 2.

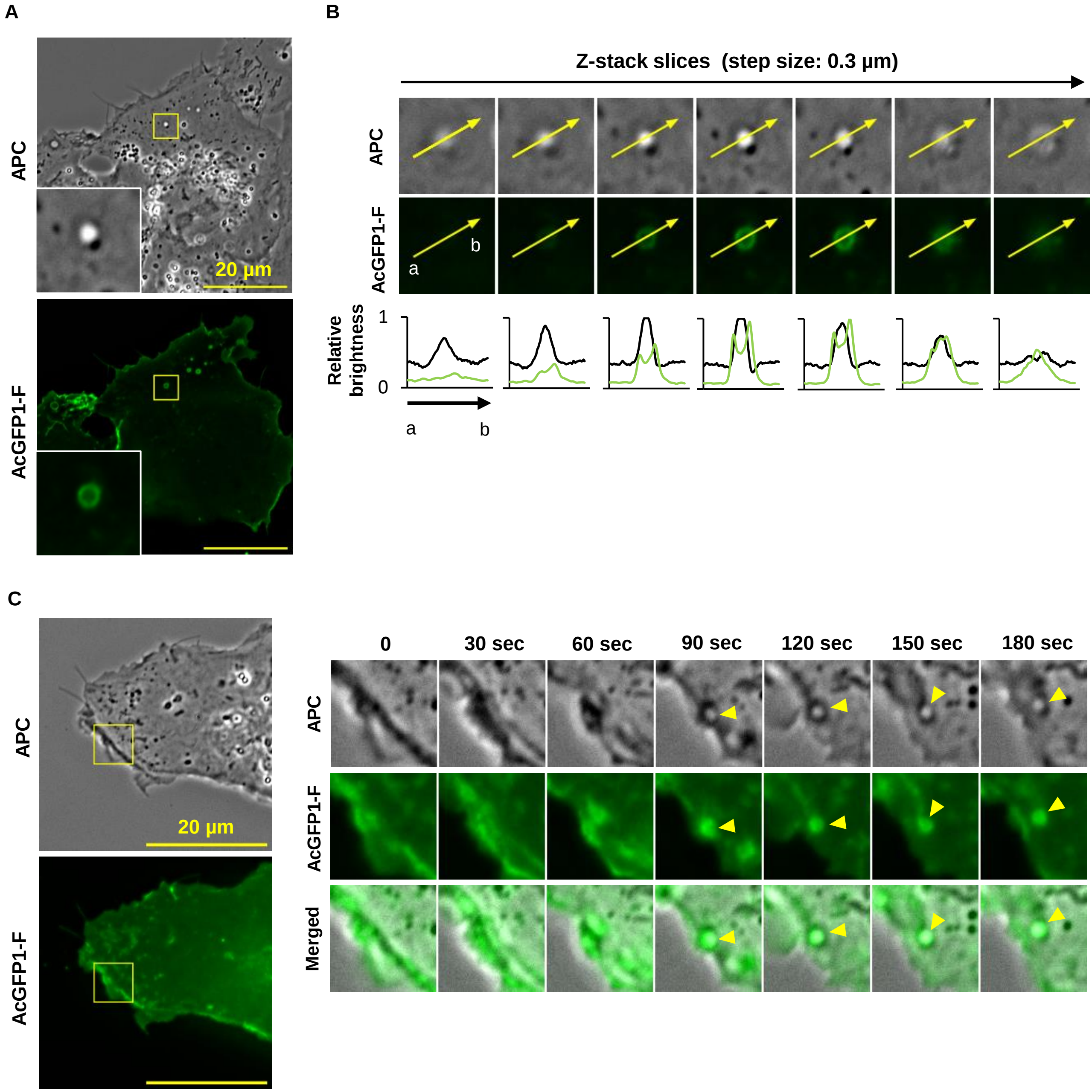

Supplemental Figure 3.

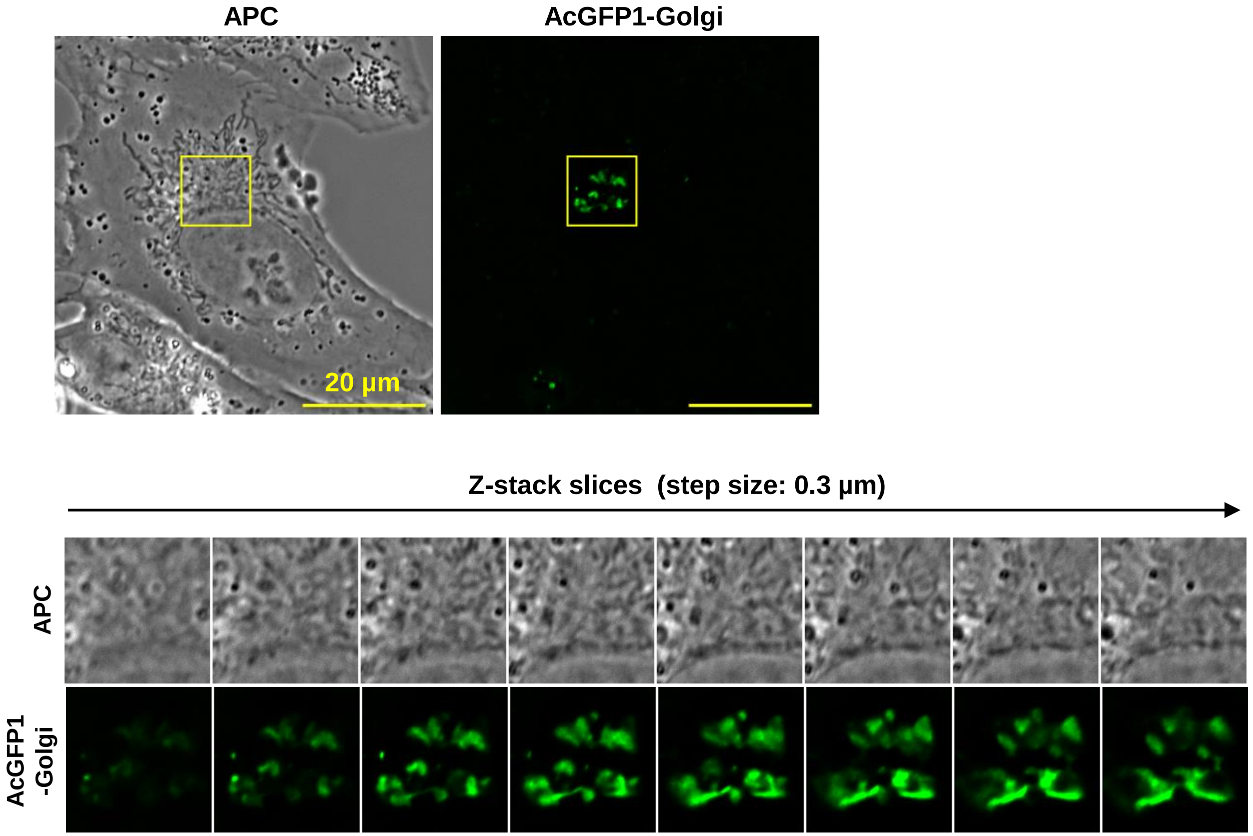

Supplemental Figure 4.

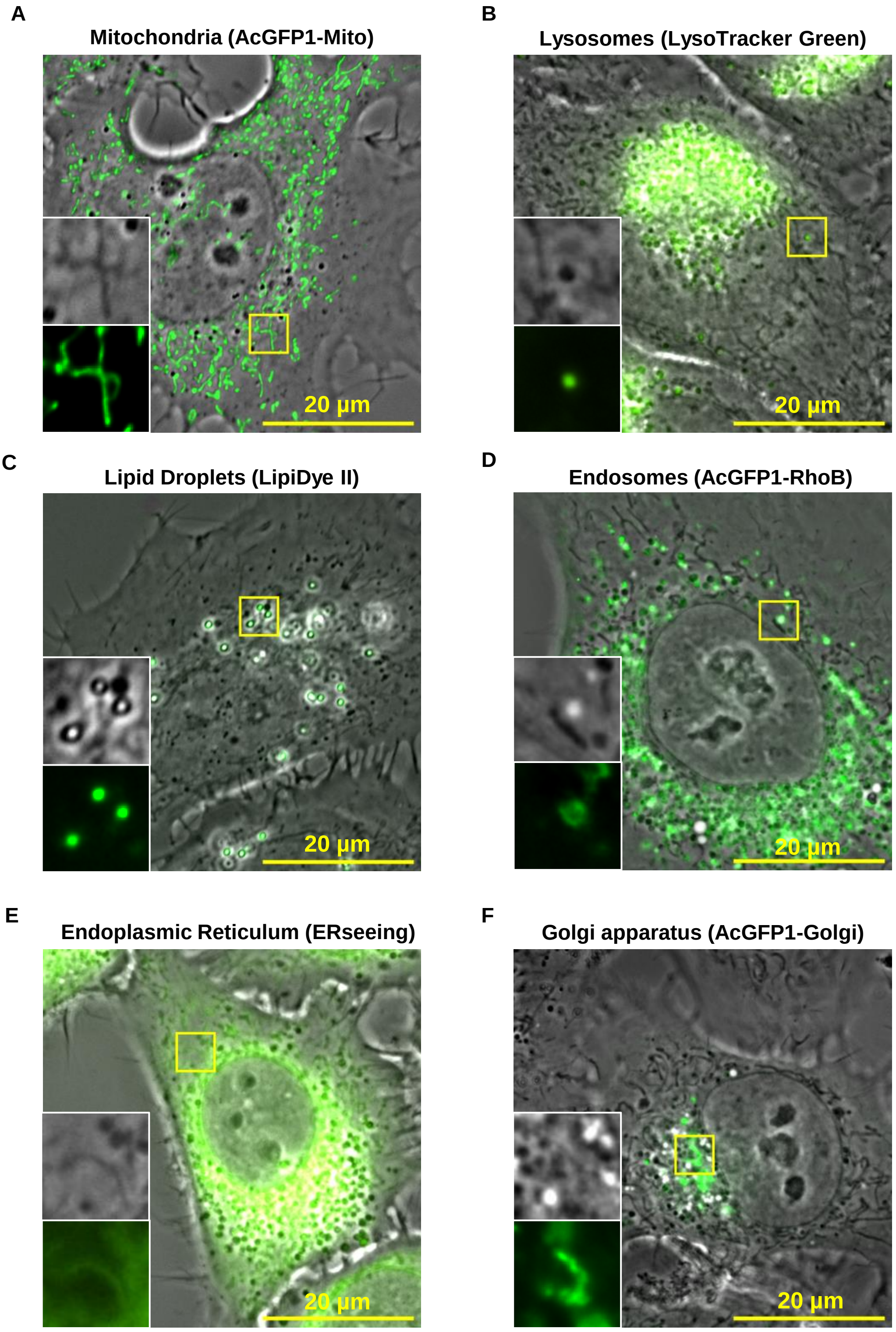

Supplemental Figure 5.

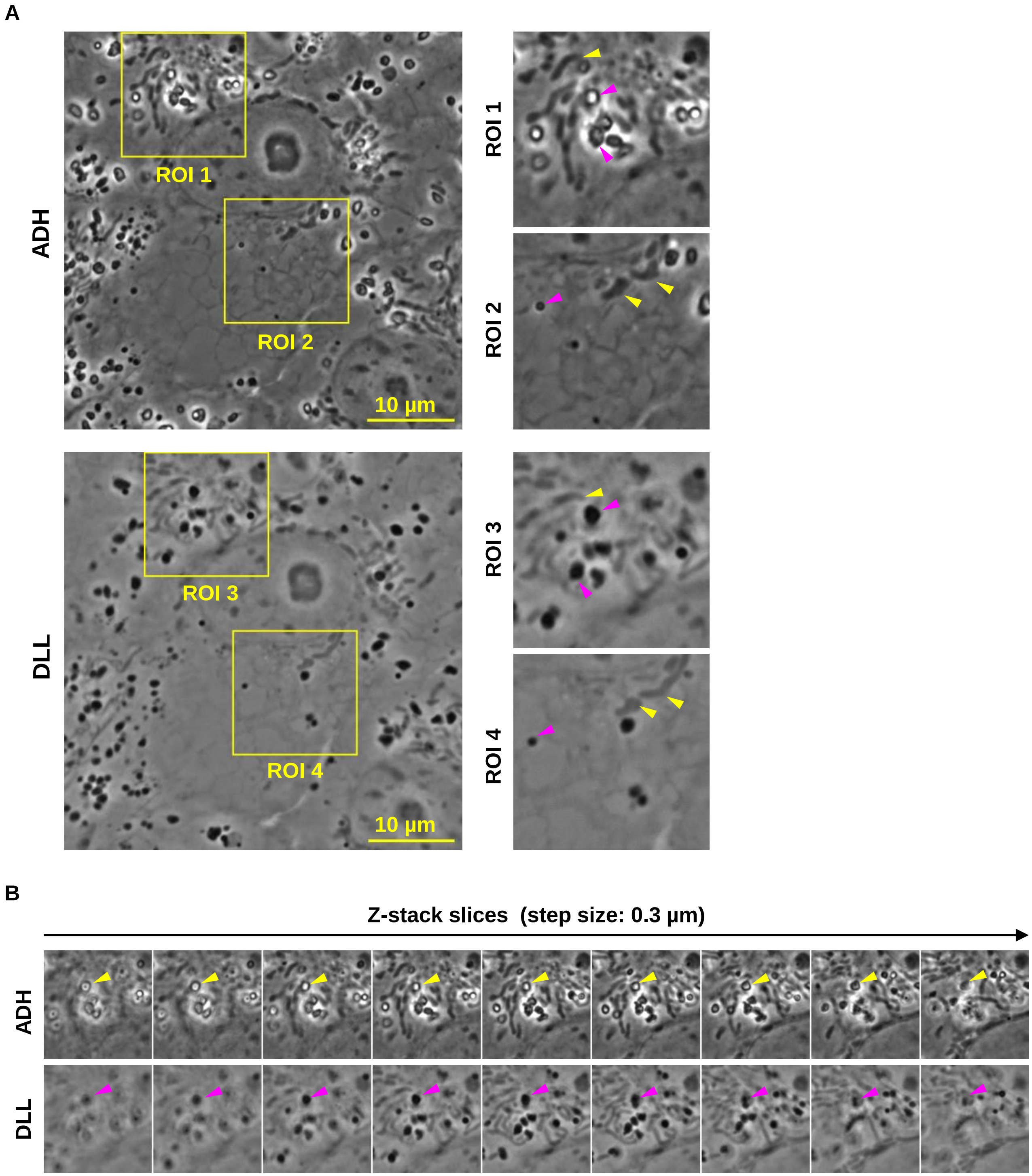

Supplemental Figure 6.

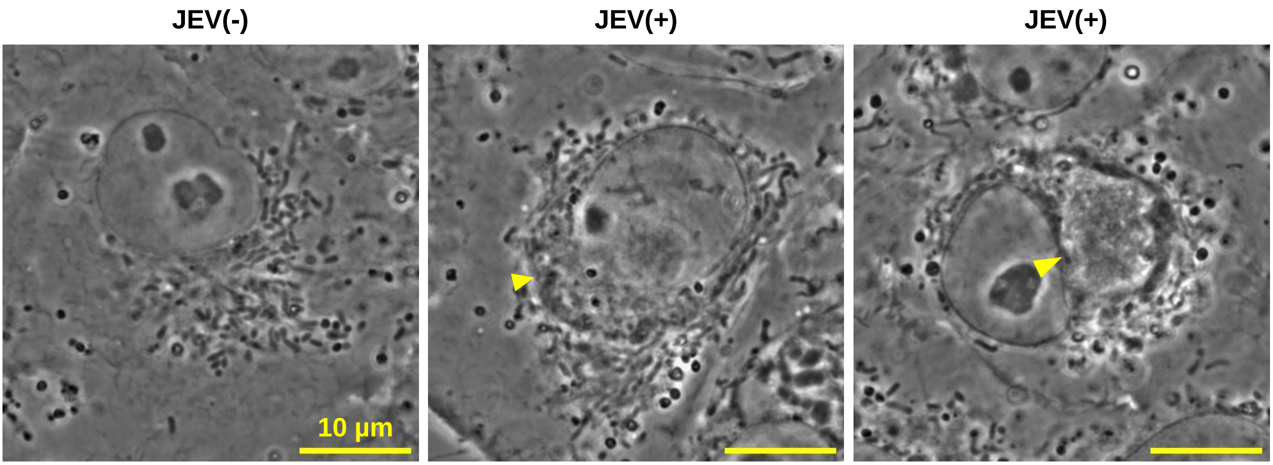

Supplemental Figure Legends.

Supplemental Fig 1. Visual characterization of organelles using Z-stack slices in the APC images. Each panel of (A)-(E) corresponds to the regions of interest depicted in Fig. 1A-E, respectively. The step size of the slices (from left to right) is 0.3 µm Histograms display the relative brightness analyzed by a plot profile along lines within the images corresponding to each slice. The maximum brightness along the lines of all the analyzed Z-stack slices in the APC or fluorescent images was normalized to 1.0. The APC histogram is depicted in black, while the fluorescence histogram is shown in green. To facilitate the comparison of object brightness between APC and fluorescent images, the look-up tables of APC images in the mitochondrion, lysosome, and ER were digitally inverted. The slices where the peaks and shapes of the histograms of relative brightness on the line in fluorescent images relatively align with those of the APC images represent the appearances of organelles nearly in focus.

Supplemental Fig 2. Additional characterization of the endocytic vesicles using Vero cells expressing the fluorescent plasma membrane markers. (A) Representative APC and fluorescent images in Vero cells expressing AcGFP1-F. (B) Z-stack images in ROI (yellow squares) drawn in (A). The step size of the slices is 0.3 µm. The histograms show the normalized brightness analyzed by a plot profile along the lines within the images corresponding to each slice. The maximum brightness along with lines of all the analyzed Z-stack slices in the APC or fluorescent images was normalized to 1.0. (C) The time series of the APC and AcGFP1-F in the ROI (yellow boxes) on the left images are shown. The yellow arrowheads show the endocytic vesicles internalized at the plasma membranes. These results confirmed that the brighter-than-cytosol vesicles in the images correspond to the endocytic vesicles. The scale bars in (A) and the left panels in (C) are 20 µm.

Supplemental Fig 3. Characterization of the Golgi apparatus in the APC live cell images. Representative APC and fluorescent images in Vero cells expressing AcGFP1-Golgi. The lower panels show Z-stack images in ROI (yellow squares) drawn in the upper image. The step size of the slices is 0.3 µm. There are no obvious structural landmarks in the APC images corresponding to the fluorescent signals. The scale bar is 20 µm.

Supplemental Fig 4. Visual characterization of various organelles in APC images in HeLa cells. Cells were labeled with AcGFP1-Mito for mitochondria (A), LysoTracker Green for lysosomes (B), LipiDye II for lipid droplets (C), AcGFP1-RhoB for endosomes (D), ERseeing for the ER (E), and AcGFP1-Golgi for the Golgi apparatus (F). The insets represent the enlarged areas of the yellow boxes with APC and fluorescence images. The scale bars in (A)-(F) are 20 µm.

Supplemental Fig 5. Image comparison between the ADH and DLL objectives. (A) Representative images of intracellular structures in the same living cells captured by the two objectives. ROI 1 with ROI 3, and ROI2 with ROI4, correspond to similar areas, respectively. Both objectives visualize intracellular structures, including mitochondria (yellow arrowheads), vesicles (magenta arrowheads), and the ER (ROI 2 and 4), with higher contrast in the ADH. The scale bar represents 10 µm. (B) Apparent differences in the appearances of vesicular structures between the ADH and DLL objectives. Z-stack slices in ROI2 (ADH) and ROI4 (DLL) with a step size of 0.3 µm are shown. Certain vesicles in the ADH images (e.g., yellow arrowheads) appear bright with darker edges, whereas in the DLL, a similar population of the vesicles (e.g., magenta arrowheads) appears dark. This dark appearance in the DLL images remains consistent across different Z-stack slices, suggesting that the differences between the two objectives are not attributed to a blurred effect.

Supplemental Fig 6. Characteristic appearance in Japanese encephalitis virus (JEV)-infected cells besides the convoluted membranes. Representative images of JEV-infected Vero cells (MOI 2.1) at 24 hpi, originated from Video 6. The yellow arrowheads represent the bulky structures induced by the JEV infection.
